# Macrophages in guided bone regeneration: potential roles and future directions

**DOI:** 10.3389/fimmu.2024.1396759

**Published:** 2024-04-26

**Authors:** Min Gou, Hang Wang, Huiqi Xie, Hongjie Song

**Affiliations:** ^1^ Department of Stomatology, Chengdu Second People’s Hospital, Chengdu, China; ^2^ State Key Laboratory of Oral Diseases and National Clinical Research Center for Oral Diseases and Department of Prosthodontics, West China Hospital of Stomatology, Sichuan University, Chengdu, China; ^3^ Laboratory of Stem Cell and Tissue Engineering, State Key Laboratory of Biotherapy and Cancer Center, West China Hospital, Sichuan University and Collaborative Innovation Center of Biotherapy, Chengdu, China

**Keywords:** macrophage, guided bone regeneration, osteoimmunomodulation, innate immunity, macrophage polarization

## Abstract

Guided bone regeneration (GBR) is one of the most widely used and thoroughly documented alveolar bone augmentation surgeries. However, implanting GBR membranes inevitably triggers an immune response, which can lead to inflammation and failure of bone augmentation. It has been shown that GBR membranes may significantly improve *in vivo* outcomes as potent immunomodulators, rather than solely serving as traditional barriers. Macrophages play crucial roles in immune responses and participate in the entire process of bone injury repair. The significant diversity and high plasticity of macrophages complicate our understanding of the immunomodulatory mechanisms underlying GBR. This review provides a comprehensive summary of recent findings on the potential role of macrophages in GBR for bone defects in situ. Specifically, macrophages can promote osteogenesis or fibrous tissue formation in bone defects and degradation or fibrous encapsulation of membranes. Moreover, GBR membranes can influence the recruitment and polarization of macrophages. Therefore, immunomodulating GBR membranes are primarily developed by improving macrophage recruitment and aggregation as well as regulating macrophage polarization. However, certain challenges remain to be addressed in the future. For example, developing more rational and sophisticated sequential delivery systems for macrophage activation reagents; addressing the interference of bone graft materials and dental implants; and understanding the correlations among membrane degradation, macrophage responses, and bone regeneration.

## Graphical Abstract

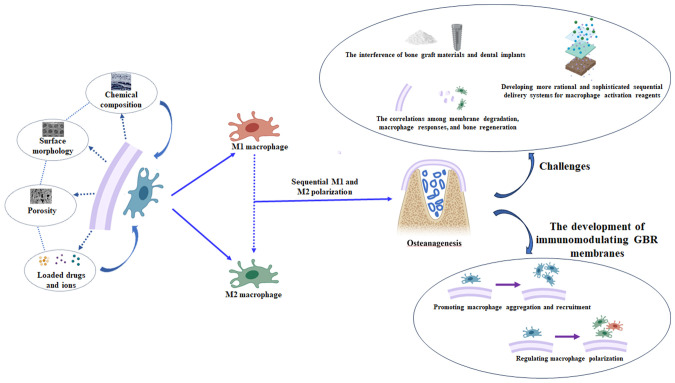

## 1 Introduction

Guided bone regeneration (GBR) is one of the most widely used alveolar bone augmentation surgeries owing to its ease of operation and reliable outcomes (1). In GBR, a barrier membrane is placed above the bone defect. This isolates fast-growing soft tissue cells, allowing slow-growing osteoblasts to preferentially occupy the defect and promote bone regeneration (2). Originally, the GBR membrane solely served as a physical barrier to prevent soft tissue penetration and provide space for osteogenesis (3, 4). However, increasing evidence suggests that GBR membranes are biologically active in bone regeneration, affecting osteoblast differentiation and stem cell recruitment (5, 6). Previous studies on GBR membrane materials mostly focused on their physical properties, antibacterial activity, and direct effects on osteoblasts and stem cells, or emphasized materials that do not elicit immune system reactions to ensure “immune safety” (7). However, inconsistent results between *in vitro* and *in vivo* conditions indicate a lack of comprehensive consideration of the immune response (8). Implantation of GBR membranes inevitably triggers a material-dependent inflammatory response, which is a significant determinant of regenerative outcomes that can lead to surgical failure (9). Bone-forming and immune cells interact within a shared microenvironment (10), with the local microenvironment, particularly the immune microenvironment, playing a key role in regulating osteogenesis (11). Therefore, it is crucial to consider immunomodulatory properties in the design of GBR membranes.

Macrophages play a crucial role in the immune response to biomaterials and exhibit significant diversity and high plasticity (12, 13). They can directly influence tissue regeneration by secreting cytokines and chemokines and participate in the entire process of damage repair (7, 14). The reduction or depletion of macrophage infiltration in GBR membranes significantly impedes bone regeneration (6, 15), and the status and sequence of macrophage polarization markedly determine the outcomes of GBR (7, 16, 17). Moreover, GBR membranes can influence the recruitment and polarization of macrophages (5, 6, 16), potentially serving as potent immunomodulators rather than traditional barrier, thereby significantly enhancing *in vivo* outcomes (16). However, the precise role of macrophages in GBR and the modulation of macrophages by GBR membranes remain unclear.

A previous review only summarized the role of collagen membranes in immune regulation (12), and most references in the literature are limited to *in vitro* experiments or certain subcutaneous and intramuscular implantation experiments, which may not accurately replicate the actual conditions of bone defect repair in situ. Therefore, in this review, we comprehensively summarize the effects of macrophages on bone regeneration in GBR, the modulation of macrophages by GBR membranes in bone defects in situ, and the current experimental progress in the development of immunomodulating GBR membranes. Additionally, we discuss future directions that may offer new insights into alternative strategies for accelerating bone healing in GBR. For instance, developing more rational and sophisticated sequential delivery systems for macrophage activation reagents, modulating the immune microenvironment by regulating the degradation of GBR membranes, and integrating the immune properties of dental implants and bone graft materials to synergistically promote bone healing.

## 2 Effects of macrophages on GBR

**Figure 1:**
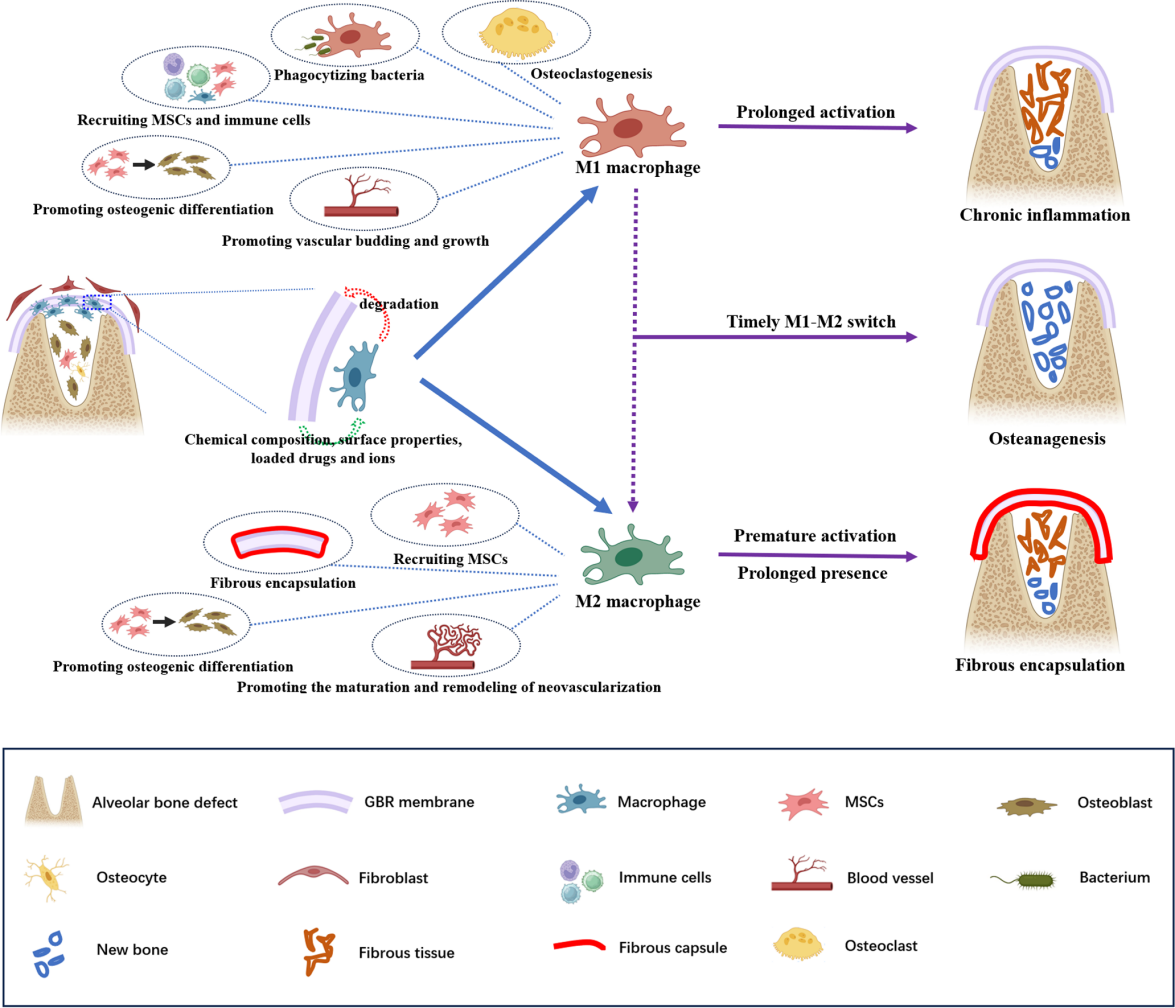
Schematic illustration of the potential roles of macrophages in guided bone regeneration of bone defects. A barrier membrane is used to prevent fibroblasts from growing into the defect but its implantation inevitably triggers an immune response. The chemical composition, surface properties, loaded drugs and ions of the GBR membrane affect the recruitment and polarization of macrophages, and macrophages may play a critical role in membrane degradation. The sequential polarization of macrophages is pivotal for the outcome of GBR. In particular, M1 macrophage polarization contributes to phagocytizing bacteria, recruiting MSCs and additional immune cells, promoting osteogenic differentiation and vascular budding and growth, and mediate osteoclastogenesis,. Polarized M2 macrophages participate in bone repair by promoting the recruitment and osteogenic differentiation of MSCs and the maturation and remodeling of neovascularization. Moreover, they also contribute to fibrous encapsulation formation. An effective and timely M1–M2 switch can enhance bone formation. By contrast, the prolonged activation of M1 macrophages will lead to chronic inflammation without osteoanagenesis; premature M2 macrophage polarization and prolonged presence may result in fibrous encapsulation around the GBR membrane, which blocks the interaction between the GBR membrane and bone tissue, thus inhibiting bone healing.

Innate immunity is activated after implanting GBR membranes, with macrophages being one of its most important functional components (18–20). Macrophages play a pivotal role in maintaining physiological bone homeostasis and modulating foreign body reactions to biomaterials (12, 21), and their polarization and spatiotemporal distribution may reflect the inflammatory status (8, 12). We hypothesize that the spatiotemporal expression profile of macrophages may indicate the outcomes of GBR (**Figure 1**).

Macrophages are heterogeneous and highly plastic cells that can be broadly characterized into two phenotypes: M1 and M2 (8). Classically activated M1 macrophages are generally considered proinflammatory, mediating inflammatory responses and osteoclastogenesis. In contrast, selectively activated M2 macrophages, known as anti-inflammatory or proregenerative macrophages, are responsible for immunomodulation and tissue remodeling (8, 12, 22).

Macrophages affect bone formation mainly by secreting various cytokines and chemokines. Within 24 h of biomaterial implantation, inflammation reaches its peak, during which M1 macrophages contribute to defending against microbes, amplifying inflammation, and attracting additional immune cells through phagocytosis and secretion of proinflammatory factors (23). It was traditionally believed that proinflammatory M1 macrophages and inflammatory reactions were detrimental to bone regeneration (8). However, increasing evidence suggests that early inflammatory stages and transient M1 macrophage polarization are crucial for bone regeneration in GBR (24, 25). Proinflammatory cytokines secreted by M1 macrophages recruit mesenchymal stem cells (MSCs) to the injury site and promote their proliferation and differentiation, initiating osteoanagenesis (26). Furthermore, M1 macrophages can recruit vascular endothelial progenitor cells to the wound site (16, 27) and promote vascular budding and growth by secreting several angiogenic factors (e.g., vascular endothelial growth factor [VEGF]), initiating angiogenesis and providing nutrients and oxygen for bone regeneration (28–30).

As the tissue insult resolves, M2 macrophages contribute to bone regeneration and remodeling by secreting anti-inflammatory factors, recruiting MSCs to bone defects, and promoting osteogenic differentiation (6). Additionally, M2a macrophages secrete platelet-derived growth factor BB to facilitate endothelial anastomoses of neovascularization and maintain the stability of neovascularization. Meanwhile, M2c macrophages participate in vascular remodeling by secreting matrix metalloproteinase 9 (28–30).

Despite the recognized pro-healing effects, premature M2 macrophage polarization and prolonged presence may be harmful to bone repair. Activated too early, M2 macrophages may actually inhibit healing and hinder vascularization by blocking VEGF function (24). Furthermore, premature or prolonged M2 macrophages secrete a large amount of fibrogenic cytokines, promoting the recruitment and proliferation of fibroblasts. This results in fibrous encapsulation around the GBR membrane, obstructing the interaction between the GBR membrane and bone tissue (25).

Notably, M1 macrophages must transition into M2 macrophages at the appropriate time. Effective and timely phenotype conversion of M1 macrophages can lead to the release of osteogenesis-enhancing cytokines by M2 macrophages, thus enhancing bone formation (8). Conversely, if M1 macrophages fail to switch to M2 macrophages in time, prolonged activation of M1 macrophages will continue to secrete proinflammatory cytokines, resulting in chronic inflammation without osteoanagenesis (24, 31). Furthermore, prolonged M1 polarization may increase the release of fibrosis-enhancing cytokines from M2 macrophages, contributing to the formation of fibrous encapsulation (8).

It is important to note that the classification of macrophages represents a simplification of the complex *in vivo* scenario. The macrophage population comprises a mixture of macrophage phenotypes at any given time, with macrophages often expressing both M1 and M2 markers, making it challenging to distinguish between M1 and M2 phenotypes (32). This variability may be attributed to changes in stimuli. For instance, experiments have shown that the cytokines secreted by lipopolysaccharide (LPS)-stimulated macrophages may transition from proinflammatory to anti-inflammatory factors when the macrophages become Toll-like receptor-tolerant (33–35). Similarly, M2 macrophages can be induced to transition into M1 macrophages upon exposure to interferon-gamma (IFN-γ) or LPS. However, the mechanisms underlying macrophage phenotypic plasticity remain unclear. In summary, it is the pattern of macrophage switching that determines bone regeneration rather than a specific macrophage phenotype. Therefore, further studies are needed to better understand the pattern of macrophage transformation and its impact on GBR.

## 3 Different GBR membrane characteristics affect macrophage behavior

**Figure 2:**
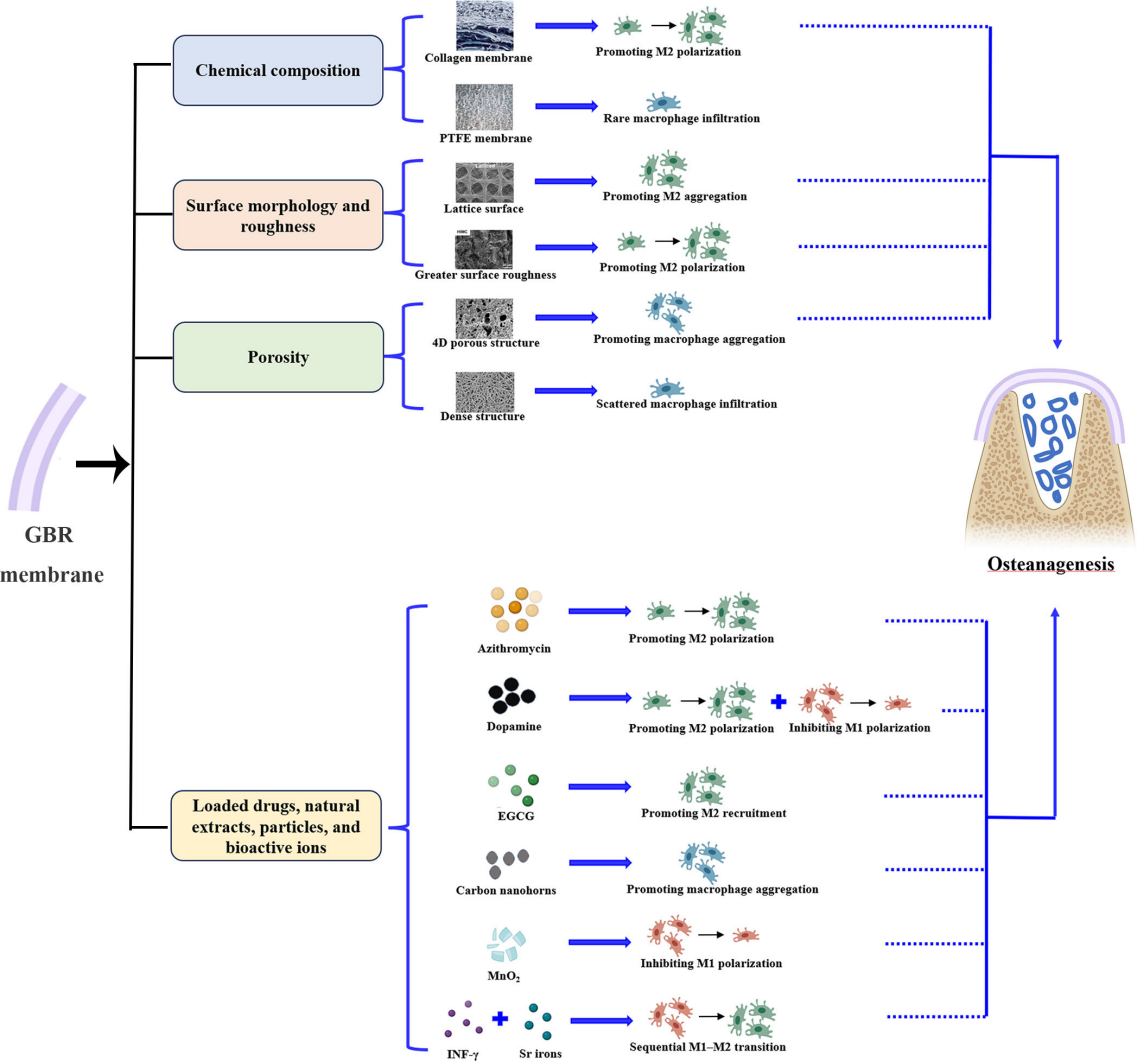
GBR membranes promote bone regeneration by affecting macrophage behavior. The chemical composition, surface properties, porosity, loaded drugs and ions of GBR membranes may modulate the aggregation, recruitment, and polarization of macrophages, thereby improving GBR outcomes.

In GBR, macrophages aggregate within the membrane compartments and at the top of the bone defect, close to the membrane (27, 36). Therefore, we speculate that the behavior of macrophages is influenced by the GBR membrane. Indeed, it has been reported that the composition, surface properties, porosity, loaded drugs and ions of GBR membranes may modulate the aggregation, recruitment, and polarization of macrophages (**Figure 2**, **Table 1**) (5–7, 15–17, 37, 38).

**Table 1:** Immunomodulating GBR membranes improve *in-situ* bone regeneration outcomes through modulating macrophage behavior.

studies	Animal models	Types of bone defects	GBR membranes	Macrophage behavior and bone regeneration outcomes
Kasai et al. (15)	rat	Calvarial bone defect	PTFE membrane; PTFE membrane loaded with carbon nanohorns	Macrophage infiltration was rarely observed in nonabsorbable PTFE membrane. The loading of carbon nanohorns improved macrophage infiltration, thereby enhancing bone regeneration.
Liu et al. (6)	rat	Calvarial bone defect	Collagen membrane; 4D membrane; 4D-PDA membrane; E-PDA membrane	Macrophage infiltration was rare and distributed thinly in collagen membrane during early stages, but it became abundant when the membrane degraded into fragments; Compared with the dense electrospun membrane, the 4D porous structure provided more space for macrophages and promoted macrophage infiltration. Dopamine coating on GBR membrane facilitated M2 macrophage transformation and inhibited M1 macrophage polarization; 4D porous structure and dopamine coating accelerated angiogenesis and osteogenesis.
Jin et al. (37)	rat	Calvarial bone defect	PFCH membranes with random, aligned, and latticed surface topologies	The lattice surface was more conducive to the recruitment of macrophages and significantly upregulated the expression levels of M2 macrophage marker genes in the osteogenic microenvironment compared to random or aligned surface topologies, which facilitated growth factor secretion and osteoblast differentiation, thus inducing bone regeneration *in vivo*.
Xuan et al. (7)	rat	skull defect	Collagen membrane; EMC membrane; HIMC membrane	HIMC membrane resembling natural bone surface, with an ordered structure and greater surface roughness, promoted M2 macrophage polarization, thus improving GBR outcomes.
Mathew et al. (17)	rat	Calvarial bone defect	PCL-CaP membrane; PCL-CaP membrane loaded with azithromycin	Compared to PCL-CaP membrane, the loading of azithromycin promoted the early switch of macrophages into proregenerative M2 subtype and maintained a lower M1/M2 ratio until the late stage of bone repair. This resulted in better bone repair outcomes.
Chu et al. (5)	rat	Calvarial bone defect	Collagen membrane; EGCG modified collagen membrane;	The modification with EGCG promoted M2 macrophage recruitment within the GBR membrane and bone defects, which facilitated growth factor secretion and osteoblast differentiation, thus inducing bone regeneration *in vivo*.
Liu et al. (38)	rat	Mandibular bone defect	HP membrane; HP@ 2%Mn membrane	The loading of MnO_2_ was capable of catalyzing the decomposition of H_2_O_2_, decreasing M1 polarization of macrophages in bone defects and improving bone repair outcomes.
Yang et al. (16)	rat	Calvarial bone defect	SIS membrane; SIS/SrHA membrane; SIS/IFN-γ membrane; SIS/SrHA/IFN-γ membrane	IFN-γ was released in bursts from membranes and stimulated transient M1 macrophage polarization during the early phase; whereas, the sustained release of strontium ions promoted M2 polarization during later stages. This resulted in sequential M1–M2 transformation and a significantly higher M2/M1 ratio, which strongly promoted vascularization and bone regeneration in situ.

PTFE: Polytetrafluoroethylene; PGS: Poly (glycerol sebacate); PCL: Polycaprolactone; PDA: Polydopamine; E-PDA membrane: PDA-coated electrospun PGS membrane; 4D membrane: The 4D-morphing membrane composed of 3D-Printing PGS/PCL construct and electrospun PGS membrane; 4D-PDA membrane: PDA-coated 4D membrane; PFCH: Poly (lactate-co-glycolate)/fish collagen/nano-hydroxyapatite; EMC: Extrafibrillarly mineralized collagen; HIMC: Hierarchical intrafibrillarly mineralized collagen; CaP: Calcium phosphate; HP: Membranes made of a combination of hydroxyapatite nanowires (HAp NWs) and polylactic acid (PLA); SIS: Small intestinal submucosa; SrHA: Strontium-substituted nanohydroxyapatite; IFN-γ: Interferon-gamma.

In terms of chemical composition, the material source and processing method can influence the response of macrophages to the material. For example, as a representative of nonabsorbable membranes, macrophage infiltration has been rarely observed in polytetrafluoroethylene membranes (15). Conversely, as a representative of absorbable membranes, although macrophage infiltration is rare and thinly distributed in collagen membranes during the early stages, it is abundant with an M2-dominant profile when the membranes degrade into fragments (6). Furthermore, chemical crosslinking of collagen with carbodiimide has been shown to result in a shift from an M2-dominant profile to an M1-dominant profile in abdominal wall repair (39). This suggests that the processing method of materials should be considered in future research on osteoimmunomodulation.

with regard to surface morphology and roughness, the lattice surface is more conducive to macrophage recruitment and significantly upregulates the expression levels of M2 macrophage marker genes in the osteogenic microenvironment compared to random or aligned surface topologies (37). Surfaces resembling natural bone, with an ordered structure and greater surface roughness, promote macrophage polarization toward M2 during the early stages of bone repair (7).

In terms of porosity, 4D porous structures promote macrophage infiltration by providing more space for macrophages. Conversely, macrophages are only recruited to the surface of the electrospun film and scattered between the membrane and bone defect because of the dense internal structure (6). Additionally, an *in vitro* experiment showed that larger pore size and higher porosity promoted M2 polarization of macrophages. This finding may provide new insights for studying bone defect repair *in situ* (40).

With regard to loaded drugs, natural extracts, particles, or bioactive ions, the release of azithromycin promotes the timely transformation of macrophages into proregenerative M2 macrophages during the early stage of bone regeneration and exhibits a lower M1/M2 ratio during the late stage of bone repair (17). Dopamine coating on GBR membranes facilitates M2 macrophage transformation and inhibits M1 macrophage polarization (6). Chu et al. (5) modified collagen membranes with epigallocatechin gallate (EGCG) and discovered that it promotes M2 macrophage recruitment within the GBR membrane and bone defects. Loading of carbon nanohorns improves macrophage infiltration during early bone repair (15), and loading of MnO_2_ decreases M1 macrophage polarization in bone defects (38). IFN-γ is released in bursts from membranes and stimulates transient M1 macrophage polarization during the early phase, whereas the sustained release of strontium ions promotes M2 polarization during later stages, thus achieving sequential M1–M2 transitions (16).

## 4 Development of an immunomodulating GBR membrane

As the importance of immunomodulatory properties in GBR membrane design has received increasing attention, developing immunomodulating GBR membranes to regulate a favorable immune environment has become a new potential strategy to accelerate bone healing (12, 41). Considering the role of macrophages in GBR, GBR membranes with immunomodulatory abilities are mainly developed by addressing two aspects. (**Table 1**).

One aspect involves the improvement of macrophage recruitment and aggregation. Kasai et al. (15) loaded carbon nanohorns onto polytetrafluoroethylene membranes to promote macrophage aggregation, which could aid in bone healing. Chu et al. (5) modified collagen membranes with EGCG and Jin et al. (37) prepared poly (lactate-*co*-glycolate)/fish collagen/nano-hydroxyapatite films with latticed surfaces to enhance the recruitment of M2 macrophages in bone defects. This facilitated growth factor secretion and osteoblast differentiation, thus inducing bone regeneration *in vivo*. The dense surfaces of electrospun membranes hinder macrophage infiltration. To overcome this disadvantage, Liu et al. (6) developed a GBR membrane with a 4D porous structure to provide more space for infiltrating cells, thereby enabling early and long-term recruitment of macrophages, which could actively participate in bone repair.

Another aspect involves the modulation of the bone immune microenvironment by regulating macrophage polarization. One approach is to promote and/or inhibit M2 and M1 subtype polarization, respectively. Mathew et al. (17) loaded azithromycin onto polycaprolactone membranes, which promoted the early switch of macrophages into proregenerative M2 macrophages and maintained a lower M1/M2 ratio until the late stage of bone repair, resulting in improved bone repair outcomes. Xuan et al. (7) prepared a hierarchical intrafibrillarly mineralized collagen membrane with a nanostructured surface morphology similar to that of natural bone, regulating M2 macrophage polarization and thus improving GBR outcomes. MnO_2_, capable of catalyzing the decomposition of H_2_O_2_,was used to modify GBR membranes, decreasing M1 macrophage polarization in bone defects and improving bone repair outcomes (38). In another study, 4D porous GBR membranes were coated with dopamine, which simultaneously promoted M2 macrophage polarization and inhibited M1 macrophage polarization, thus accelerating angiogenesis and osteogenesis (6). The other approach is to promote sequential M1 and M2 polarization. IFN-γ released in bursts was used to stimulate brief and excessive M1 macrophage polarization during the early stage of bone repair, and sustained release of strontium ions was used to stimulate M2 macrophage polarization during the late stage of bone repair. This resulted in sequential M1–M2 transformation and a significantly higher M2/M1 ratio, strongly promoting vascularization and bone regeneration *in situ* (16).

Although all of the above methods promoted bone regeneration in GBR, it is worth noting that depletion of M1 macrophages can impair fracture healing (42), and excessive M2 polarization may lead to pathological fibrosis, delaying bone healing (25). Therefore, inducing an appropriate immune environment using biomaterials at a specific time is crucial for bone healing. Precise sequential macrophage polarization may be the focus of future research, rather than blindly promoting M2 macrophage or inhibiting M1 macrophage polarization.

## 5 Future directions for immunomodulating GBR membranes

Although immunomodulating GBR membranes are gradually gaining attention, many issues need to be addressed. First, current experiments are limited to small animal studies, necessitating further *in vivo* and *in situ* experiments to extrapolate the research findings to humans.

Second, in addition to the factors affecting the recruitment and polarization of macrophages mentioned above, the hydrophilicity and charge of biomaterials also influence macrophage behavior. Hydrophilic and negatively charged biomaterials promote M2 macrophage polarization, whereas hydrophobic and positively charged biomaterials promote M1 macrophage polarization (43). These findings indicate directions for potential strategies for developing immunoreactive GBR membranes.

Sequential polarization of macrophages is critical for the outcome of GBR (24). However, several key aspects remain unclear, including the optimal timing of the M1–M2 transformation, the appropriate M1/M2 ratio and duration for different stages of bone healing, and the mechanism underlying the M1–M2 transformation. Additionally, a major limitation of current sequential drug delivery systems is the lack of distinct separation between the release stages. Many systems that successfully achieve slow controlled delivery of M2 activation reagents do not entirely prevent their release within the initial days. This early release, even in small doses, may induce mixed phenotypes that impair crucial M1 activity (24). Therefore, there is a need for more rational and sophisticated sequential drug delivery systems.

Furthermore, the role of macrophages in GBR has been poorly studied in elderly patients and patients with systemic diseases (e.g., diabetes, systemic lupus erythematosus, and Sjogren’s syndrome) and thus dysfunctional immune systems (44–47). These vulnerable populations should be addressed in future studies.

Notably, when dental implant surgery and GBR are performed simultaneously, the barrier membrane, bone graft material, and dental implant are spatially very close, resulting in a more complex immune microenvironment. Certain materials may maintain macrophage homeostasis and promote bone regeneration, whereas others may elicit serious foreign body reactions, ultimately leading to bone integration failure (12, 48). Modifications of dental implants and bone graft materials can regulate the local immune microenvironment; for example, loading cytokines or altering the forms and surface morphologies of materials (49–52). Therefore, integrating the immune properties of dental implants and bone graft materials to synergistically promote bone healing may be an alternative strategy to improve the outcome of GBR.

Furthermore, the barrier function integrity of the absorbable GBR membrane must correspond to the rate of bone regeneration (53). If the GBR film degrades too quickly, its barrier function may be lost prematurely, thus impeding bone regeneration (6). Conversely, materials that degrade slowly may hinder macrophage spreading onto the surface, leading to the formation of foreign body giant cells (FBGCs) and subsequent fibrous encapsulation (54). This encapsulation prevents direct interactions between the GBR membrane and the surrounding environment. Although the material can persist in the body for sufficient periods, it also isolates the interaction between the GBR membrane and osteoblasts, ultimately impeding bone regeneration (12).

Macrophages may play a crucial role in membrane degradation (55). When exposed to small particles (<5 μm), macrophages mainly participate in degradation through phagocytosis. However, when exposed to larger biomaterials (>10 μm), macrophages coalesce to form FBGCs. This process is induced by interleukin-13, primarily released by T helper 2 cells (9, 54–56). FBGCs exhibit enhanced phagocytic capability by expanding the contact area with the material (55). Moreover, they further degrade materials by releasing reactive oxygen species and degradative enzymes, including matrix metalloproteinases (57). In addition to macrophages, neutrophils are actively involved in membrane degradation by releasing hydrolytic enzymes and oxidative compounds (58).

Moreover, the degradation of collagen membranes may actively drive the transformation of proregenerative macrophages. Chemoattraction by products degraded from collagen could contribute to the recruitment and polarization of M2 macrophages (59, 60). When degradation is delayed or prevented by crosslinkers, polarization of M2 macrophages is inhibited, thereby impeding tissue regeneration (39). Therefore, modulating the immune microenvironment by regulating GBR membrane degradation emerges as a plausible strategy to manipulate GBR outcomes.

## 6 Conclusion

The implantation of GBR membranes inevitably activates innate immunity, in which macrophages play a vital role, participating in the entire process of bone healing. The composition, surface properties, loaded drugs, and ions of the GBR membrane affect the recruitment and polarization of macrophages. The sequential polarization of macrophages is pivotal for the outcome of GBR. In particular, early transient M1 macrophage polarization is critical for bone regeneration as it initiates acute inflammation and angiogenesis. M1 macrophages must transition into M2 macrophages at the appropriate time; otherwise, foreign body reactions become unbalanced, impeding bone healing. Polarized M2 macrophages participate in bone repair by promoting the recruitment and osteogenic differentiation of stem cells and the maturation and remodeling of neovascularization. However, the extent and duration of macrophage polarization and the precise timing of the M1–M2 macrophage switch remain unclear. Currently, the development of immunomodulating GBR membranes mainly focuses on regulating the recruitment and polarization of macrophages. Therefore, one of the future directions may involve developing more rational and sophisticated sequential delivery systems for macrophage activation reagents. Additionally, modulating the immune microenvironment by regulating GBR membrane degradation and integrating the immune properties of dental implants and bone graft materials to synergistically promote bone healing may be serve as the alternative strategies to improve the outcome of GBR. In summary, improvements in GBR membranes based on immunomodulation hold great potential for optimizing bone regeneration outcomes.

## Author contributions

MG: Investigation, Writing – original draft. HW: Writing – original draft. HX: Writing – original draft, Writing – review & editing. HS: Funding acquisition, Writing – original draft, Writing – review & editing.
